# Early postoperative use of CPAP reduces need for unplanned IPPV in elective vascular patients

**DOI:** 10.1186/cc14299

**Published:** 2015-03-16

**Authors:** H Kennedy, J Navein, J Seidel

**Affiliations:** 1 Doncaster Royal Infirmary, Sheffield, UK

## Introduction

Respiratory failure is a well-known complication of aortic aneurysm surgery. We describe the impact of a protocol, using CPAP after elective surgery to reduce the need for unplanned invasive ventilation.

## Methods

In 2012 we introduced a CPAP protocol for patients undergoing elective aortic aneurysm surgery, either open (AAA) or as an endovascular repair (EVAR). According to pre-existing risk factors (see Table 1) and arterial blood gas analysis in the anaesthetic room, they were assigned to two alternative options on the ITU: prophylactic CPAP for 9 hours in each of the first two postoperative nights or oxygen via face mask. CPAP was applied at any time in the patients stay, if their P/F ratio dropped below 40. Criteria to stop CPAP were also predefined. Previously, CPAP was initiated at the discretion of nursing staff, P/F ratios were not utilised.

**Table 1:** Criteria for the use of prophylactic CPAP.

Current smoker
FEV1: <1.5 l/minute
Poor exercise tolerance (<100 yards) due to chest problems
SpO_2_ <92% on air
SpO_2_ <92% on FiO2 >0.4 in theatre

## Results

We compare patient cohorts in the years 2010 and 2011 (pre protocol) with 2013 and 2014 (post protocol). **Results **are reported as the split between open surgery and endovascular repair. Table 2 presents requirements for invasive ventilation (IPPV) and length of stay (LOS) for both patient groups.

**Table 2:** IPPV requirements and length of stay data 2010 to 2011 2013 to 2014.

	2010 to 2011			2013 to 2014		
**IPPV**	**LOS ITU (days)**	**LOS hospital (days)**	**IPPV**	**LOS ITU (days)**	**LOS hospital (days)**	**EVAR**

2/67 (3%)	2	6	1/77 (1.3%)	2	5	AAA
10/46 (21.7%)	5	9	6/37 (16.2%)	5	12	

## Conclusion

There is a clear reduction in the need for unplanned IPPV in both patient groups. An audit in 2013 showed incomplete protocol adherence in the ITU, therefore benefits may be underestimated.

